# Feature Weight Driven Interactive Mutual Information Modeling for Heterogeneous Bio-Signal Fusion to Estimate Mental Workload

**DOI:** 10.3390/s17102315

**Published:** 2017-10-12

**Authors:** Pengbo Zhang, Xue Wang, Junfeng Chen, Wei You

**Affiliations:** State Key Laboratory of Precision Measurement Technology and Instruments, Department of Precision Instrument, Tsinghua University, Beijing 100084, China; zpb14@mails.tsinghua.edu.cn (P.Z.); chenjf17@mails.tsinghua.edu.cn (J.C.); youw16@mails.tsinghua.edu.cn (W.Y.)

**Keywords:** mental workload, signal fusion, n-back task, mutual information, heterogeneous bio-signals

## Abstract

Many people suffer from high mental workload which may threaten human health and cause serious accidents. Mental workload estimation is especially important for particular people such as pilots, soldiers, crew and surgeons to guarantee the safety and security. Different physiological signals have been used to estimate mental workload based on the n-back task which is capable of inducing different mental workload levels. This paper explores a feature weight driven signal fusion method and proposes interactive mutual information modeling (IMIM) to increase the mental workload classification accuracy. We used EEG and ECG signals to validate the effectiveness of the proposed method for heterogeneous bio-signal fusion. The experiment of mental workload estimation consisted of signal recording, artifact removal, feature extraction, feature weight calculation, and classification. Ten subjects were invited to take part in easy, medium and hard tasks for the collection of EEG and ECG signals in different mental workload levels. Therefore, heterogeneous physiological signals of different mental workload states were available for classification. Experiments reveal that ECG can be utilized as a supplement of EEG to optimize the fusion model and improve mental workload estimation. Classification results show that the proposed bio-signal fusion method IMIM can increase the classification accuracy in both feature level and classifier level fusion. This study indicates that multi-modal signal fusion is promising to identify the mental workload levels and the fusion strategy has potential application of mental workload estimation in cognitive activities during daily life.

## 1. Introduction

Mental workload influences human performance in the specific scene or task. In recent years, heavy workload has become the ubiquitous phenomenon that may decrease the task efficiency, threaten human health and cause serious accidents. It is important to monitor and estimate mental workload levels for some particular jobs such as pilot, soldier, crew, and surgeon. Traditional measurement uses questionnaires or mental fatigue scales such as the NASA Task Load Index (NASA-TLX) to estimate mental workload. The self-rating methods can be utilized as the standard for mental workload estimation because they have the reliability and the sensitivity. These methods are practical for clinical trials and scientific experiments. However, they are subjective and cannot record the mental workload states continuously. Therefore, the focus on physiological signals for mental workload estimation is increasing.

EEG is one of the most important physiological signals to analyze mental workload. It reflects the electrical activity of the cortex directly [1]. EEG has high temporal resolution, which is important to measure mental states continuously. Because of the sensitivity to cognitive stimuli, EEG is capable of conducting experiments for mental workload estimation. Worldwide research groups proposed their methods based on EEG with a hope that mental workload estimation could be accurate and convenient.

Many researchers developed the classifiers for mental workload estimation. Baldwin et al. designed an artificial neural network for mental workload classification based on EEG spectral analysis [2]. Dong Qian proposed Bayesian-copula discriminant classifier (BCDC) based on the copula theory and kernel density estimation to detect drowsiness during daytime [3]. Some researchers focused on EEG feature extraction. For example, Roy et al. developed an efficient mental workload estimation method by the combination of power spectral density (PSD) features, and event-related potential (ERP) features [4]. Rifai Chai et al. focused on the EEG source separation and proposed an independent component analysis method by entropy rate bound minimization analysis (ERBM-ICA) to improve the driver fatigue classification [5]. Their research demonstrates that signal processing before machine learning is also important to improve the performance of mental workload estimation. In recent years, some researchers began to try deep learning methods for cross-day mental workload estimation. Zhong Yin et al. developed an adaptive Stacked Denoising AutoEncoder (SDAE) for mental workload estimation and explained that deep learning methods might be superior in comparison with static classifiers [6]. Ryan G. Hefron et al. announced that temporal dependency of EEG was promising to improve the cross-day cognitive workload estimation [7]. They achieved a significant result in within-participant cross-day condition based on deep long short-term memory structures.

Though EEG attracts most researchers in this field, research on the other physiological signals will enhance the performance of mental workload estimation. Hoover et al. proposed a real-time detecting method based on heart rate variability (HRV), which demonstrated that HRV might be a good indicator of mental workload [8]. However, the single-modal signal has the limitation to classify different mental workload levels accurately. It is a major challenge to improve the detection accuracy based on multi-modal physiological signals [9]. Whang et al. collected EEG and ECG signals to research 3D visual fatigue using heartbeat evoked potential (HEP) based on heart-brain synchronization [10]. Florence et al. combined EEG feature vector and ECG feature vector to improve the rapid detection of mental fatigue and found that the combined feature vector can enhance the capability of the classifiers [9]. Moreover, Jagannath et al. assessed the early onset of driver fatigue using EEG, ECG, blood pressure, oxygen saturation level and surface electromyography [11]. Gergelyfi et al. measured EEG, pupil size, eye blinks, skin conductance responses of the subjects in different work memory tasks [12]. Even though many types of physiological signals have been researched to estimate mental workload, few researchers focused on the signal fusion strategy. They just found the statistical results between physiological signals and different mental workload states but neglected that the heterogeneous signal fusion may be a key to improve the performance of mental workload estimation methods.

Signal fusion methods have attracted the attention of many researchers for solving pattern recognition problems. Signal fusion is also promising to extend the application of wireless sensor networks in various fields [13]. It provides the interface to utilize the large-scale information and improves the classification results. The researchers usually divide signal fusion methods into three categories which are early fusion, intermediate fusion, and late fusion [14]. Early fusion is also named as feature level fusion which emphasizes the data combination before the classification. The final feature vector consists of the features extracted from heterogeneous signals, and early fusion should put the final feature vector into the classifier alone. However, as for late fusion, different feature vectors should be fed into the classifiers respectively, and the final prediction is the combination of the different classification results. Therefore, late fusion is also called classifier level fusion or decision level fusion. Intermediate fusion represents the method between early fusion and late fusion.

Multi-modal physiological signal fusion is promising to solve biometric pattern recognition problems. For body sensor networks, multi-sensor fusion is fundamental to the applications of health-monitoring, motion recognition and other applications of the Internet of Things [15]. Verma et al. concatenated the feature vectors based on heterogeneous physiological signals for emotion recognition and validated the effectiveness of feature level fusion [14]. Hogervorst et al. combined the information of EEG, skin conductance, respiration, ECG, pupil size and eye blinks for mental workload estimation [16]. He concatenated features for feature level fusion and used the average score for classifier level fusion. Christensen et al. combined the features of EEG, ECG, and EOG and applied ANN, SVM, and LDA to validate the fused feature vector. After feature concatenation, Yin et al. embedded the feature selection method into signal fusion, which improved the performance for mental workload estimation [17].

Though some researchers have begun to use signal fusion methods to estimate mental workload, it is still necessary to improve the fusion algorithms. They concatenated different feature vectors but did not consider the dependency and redundancy information of the features. However, beyond the information combination, information filtering is also indispensable. For solving this problem, this paper proposes interactive mutual information modeling (IMIM) for both feature level and classifier level fusion to increase the classification accuracy of different mental workload states. Mutual information is an efficient feature selection method which can utilize the dependency information and eliminate the redundancy information [18]. However, few researchers think of its potential to estimate the feature weights for signal fusion. This paper optimizes the mutual information algorithm and extends its application to solving feature level and classifier level fusion problems. Considering the complicated interaction of the features extracted from different signals, This paper propose IMIM and validate it based on the features of EEG, ECG signals. Feature level and classifier level fusion are completed based on IMIM.

The main contribution of this work is threefold: First, this study proposes interactive mutual information modeling to estimate feature weights. Second, feature level and classifier level fusion methods are developed based on the feature weights. Third, mental workload classification accuracy is improved. Because of the ability to analyze the relationship between physiological signals and mental workload states, IMIM can be utilized to develop the body sensor networks for mental workload estimation.

The remainder of this paper is structured as follows. Section 2.1 introduces data recording in the mental workload tasks. Section 2.2 summarizes the important features for mental workload estimation and explains the extraction of feature vectors for heterogeneous bio-signal fusion. Section 2.3 displays the historical evolution of mutual information and describes the derivation of the proposed objective function of interactive mutual information modeling (IMIM). Section 2.3 also introduces the development of feature level and classifier level fusion methods based on IMIM. The experiments are presented in Section 3 to evaluate the performance of the proposed method. Feature level fusion methods, classifier level fusion methods, and other mental workload estimation methods are all utilized for comparison. Finally, Section 4 gives the conclusion.

## 2. Method

### 2.1. Materials

This study invited ten subjects from Tsinghua University to take part in the experiment. They were all males and right-handed. The ages of the participants ranged from 22 to 28. All of the subjects were asked to stay away from caffeine and alcohol for at least 24 h. They were required to have enough sleep before the experiment. These restrictions were helpful to guarantee that the participants had the same baseline to start the work memory tasks.

Memory workload is an important aspect of mental workload. Memory workload can be defined as the ability to memorize and analyze short-term information [19]. Heavy memory workload will disable humanity from solving serious problems in real life. This paper uses memory workload as an example to explore mental workload estimation methods. To induce different memory workload levels, most researchers utilize several kinds of mental workload tasks based on the assumption that the harder task can cause the higher mental workload. One of the most practical tasks is the n-back task which was first designed in 1958 by Kirchner [20]. Because of its convenience and effectiveness, the n-back task has been widely used to research memory workload based on the dynamic information of letters and positions. In order to ensure the reliability and comparability of the proposed method, this study used traditional 1-back, 2-back, and 3-back position memory tasks to induce low, medium and high mental memory workload.

**Figure 1:**
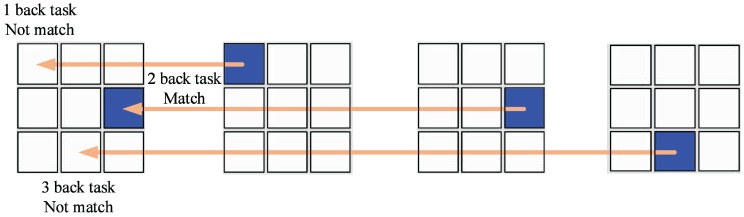
A sequence of position stimuli in 1-, 2-, 3-back tasks.

During the n-back task, as Figure 1 shows, the screen of a computer displayed a big square which consisted of nine different positions. A small blue block would appear randomly at one of the nine areas every three seconds. In the 1-back task, the subjects should compare the current position of the blue block with the preceding one. They needed to press the A key on the keyboard as quickly as possible when the 2 positions became the same. Analogously, participants should compare the current position with the one before just one in the 2-back position task and judge the previous position of the one before just one in the 3-back position task. To validate the effectiveness of the n-back task, we recorded the reaction time and the correct ratio of the subjects for statistical analysis.

This study collected multi-modal physiological signals from 3 different workloads in the experiment which consisted of the 1-back task, 2-back task, and 3-back task. The three tasks were supposed to induce low, medium and high memory workload. As Table 1 shows, before the experiment, subjects would be given 5 min to calm down and prepare for the tasks. Each session contained one task which had 200 trails of random positions. After each task, the subjects should complete the NASA Task Load Index for self-rating. Then they would be given 3 min to have a rest and prepare for the next task. The entire experiment lasted 47 min.

EEG and ECG signals were both collected during the tasks. This study used 16 channel EEG headset based on the 10–20 system with a 1000 Hz sampling rate to obtain EEG signals. The average of two ear electrodes was the reference of the EEG headset. This study used a patient monitor manufactured by Mindray company to record ECG signals and calculate the R-R intervals during the experiment. There were 600 s physiological signals during each n-back task, and the samples were extracted using 90 s signals with a 3 s step. Therefore, we obtained 171 samples from every subject in each task. The response time and the accuracy of the subjects in this experiment were recorded to analyze their performance.

### 2.2. Feature Extraction

#### 2.2.1. EEG Feature Extraction

EEG is the most important physiological signal to analyze mental workload because it reflects the cortical activities directly [21]. However, EEG is so weak that multiple kinds of noise may be induced in the recording process. The main artifacts of EEG are caused by eye blinks, muscle contraction and other devices in the measurement system [22]. ICA (independent component analysis) has been widely used to calibrate the noise sources for artifact removal [23].

This study used an EEG analysis toolbox named EEGLAB [24] for the preprocessing of EEG signals. First, EEG signal was filtered from 0.5 Hz to 100 Hz to remove the direct current voltage and high-frequency artifacts. Second, this study utilized ADJUST algorithm to remove eye blink artifacts based on ICA [25]. Third, EEG signal was segmented as 3 s epochs based on the stimuli in the n-back tasks. There were 200 epochs of every person in each task. Each sample was extracted using 30 epochs with 1 epoch step. Therefore, this study collected 5130(171×3×10) samples for mental workload estimation.

Many research groups developed their mental workload estimation algorithms and proposed numerous types of features. EEG power spectral density (PSD) features and event-related potential (ERP) features were the most effective features for mental workload estimation.

PSD features were extracted based on the concatenation of all the epochs in each sample. In recent years, Welch’s method, a periodogram spectrum estimator, has been utilized to extract EEG PSD features [26,27]. Due to the insensitiveness to the noise, we used it to extract 2 types of PSD features. These PSD features have proved their effectiveness in the previous study. First, we extracted the PSD features from 5 traditional frequency bands (δ [1∼3 Hz], θ [5∼7 Hz], α [9∼12 Hz], β [14∼31 Hz] and γ [33∼42 Hz]) and 2 expanded bands (γ1 [33∼57 Hz], γ2 [63∼99 Hz]) [2,28,29,30,31,32,33,34]. Second, the PSD features of all frequency bands from 1 Hz to 40 Hz in a 1 Hz step were calculated [35,36]. Therefore, 736(46×16) PSD features were extracted from 16 EEG channels.

ERP features have been widely used to analyze mental workload because they can reflect the EEG activity according to visual stimuli. In this study, ERP signal was calculated using the average epoch of each sample from 0 to 1000 ms after the onset of each stimulus. This study extracted 2 types of ERP features from the ERP signal based on the previous research. First, the ERP signal was down-sampled to 100 Hz, and every point of the signal might be a useful feature [16,37]. Then 101 features were extracted from each channel. Second, this study calculated the value of the wave peak, wave valley, and the corresponding frequency of the peak and valley in each channel to obtain another 4 important ERP features [38]. Therefore, this experiment extracted 1680(105×16) ERP features in total.

Considering all of the PSD features and ERP features, this study used 2416 EEG features to explore the signal fusion methods for mental memory workload estimation.

#### 2.2.2. ECG Feature Extraction

ECG has become one of the focuses for mental workload estimation. Heart rate (HR) and heart rate variability (HRV) have proved their efficiency to distinguish different mental workload levels. This study used the HRV analysis software (HRVAS) to extract numerous ECG features. HRVAS is a practical tool to extract time domain, frequency domain, time-frequency domain and nonlinear features for ECG analysis [39]. We used it to extract 103 ECG features based on each ECG sample.

### 2.3. Interactive Mutual Information Modeling

#### 2.3.1. Mutual Information

Section 2.2 has mentioned that this experiment utilized 2 EEG feature vectors and 1 ECG feature vector for mental workload estimation. Nevertheless, it is hard to estimate the importance of each feature in different feature subsets. Just combining various types of features cannot achieve satisfactory results. Research on heterogeneous signal fusion is still a challenge. Mutual information is an important index to reflect the dependency between the label vector and the feature vector [40]. Many researchers used mutual information for feature selection because it is effective to measure the performance of each feature subset. Besides its application for feature selection, it has the potential to estimate the weight of each feature for signal fusion. The preliminaries of mutual information are described as follows.

Assume that *x* represents a feature and *y* represents the label. The mutual information of *x* and *y* measures the dependency between them. Mutual information is denoted as I(x;y). The probability density functions (PDFs) and the joint PDF of the two variables are represented as P(x), P(y), P(x,y) respectively. The definition of mutual information is:

(1) $$I\left(x;y\right)=\int P\left(x,y\right)\log\frac{p(x,y)}{p(x)p(y)}dxdy$$

The entropy of variable *x* is denoted as H(x) which represents the amount of information contained in *x*. And H(x|y) represents the conditional entropy which means the increased amount of information given by variable *x* when variable *y* has been known. Mutual information can also be represented as:

(2) $$\begin{array}{c} I(x;y)=H(x)+H(y)-H(x,y)\\ I(x;y)=H(x)-H(x|y)=H(y)-H(y|x) \end{array}$$

where H(x)=−∫P(x)logP(x)dx, and H(x|y)=−∫P(x,y)logP(x|y)dxdy

Nevertheless, Equation (1) can only measure the relevance of two variables. In order to measure the transmitted information between multi-dimensional feature vector and label, McGill extended the expression of mutual information [41]:

(3) $$I\left(x;y\right)=H\left(x_{1},x_{2},\cdots x_{n}\right)+H\left(y\right)-H\left(x_{1},x_{2},\cdots x_{n},y\right)$$

where x denotes the n dimensional feature vector, and $I\left(x;y\right)=I\left(x_{1},x_{2},\cdots x_{n};y\right)$.

The joint entropy H(x) is denoted as:

(4) $$H\left(x\right)=-\int P\left(x_{1},x_{2},\cdots ,x_{n}\right)\log\frac{P(x_{1},x_{2},\cdots ,x_{n})}{P\left(x_{1}\right)P\left(x_{2}\right)\cdots P\left(x_{n}\right)}$$

**Figure 2:**
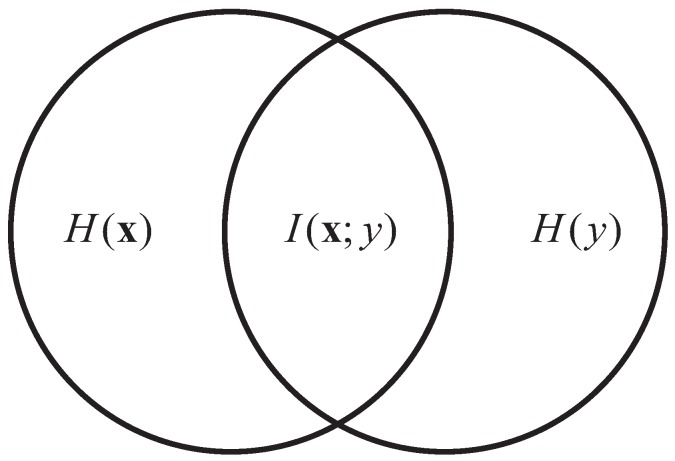
Relation of the input variables and label according to mutual information.

As Figure 2 shows, H(x) and H(y) represent the entropy of the original feature vector x and the label *y* respectively. The mutual information I(x;y) is a measure of the information shared between H(x) and H(y). Therefore, the focus of feature selection methods based on mutual information is to find the feature subset of x which can maximize the mutual information I(x;y).

Due to the complexity of the estimation of high dimensional probability density function, Equation (3) needs to be simplified for further calculation. Therefore, many research groups proposed the general equations to define the relationship between multi-variable entropy and mutual information [42,43]. Co-information which was proposed by Bell is practical to simplify the calculation based on mathematical transformation [43]. Provided that x represents the multi-variable $\left\{x_{1},x_{2},\cdots ,x_{n}\right\}$, $x^{\left(k\right)}$ represents one of the subsets of x and the dimension of $x^{\left(k\right)}$ is denoted as $|x^{\left(k\right)}|$, co-information can be defined as follows:

(5) $$I\left(x\right)=-\sum_{x^{\left(k\right)}\subseteq x}{\left(-1\right)}^{|x^{\left(k\right)}|}H\left(x^{\left(k\right)}\right)$$

Bell derived the general formulation of co-information according to Equation (5):

(6) $$I\left(x\right)=\int_{x}p\left(x\right)\log\prod_{x_{i}\subseteq x}p{\left(x_{i}\right)}^{{\left(-1\right)}^{|x_{i}|}}dx$$

In order to transfer the formulation of the mutual information given by Equation (3), Equation (5) defines the relationship between co-information and multi-variable entropy. The multi-variable entropy can be represented by a symmetrical formulation based on Equation (5):

(7) $$H\left(x\right)=-\sum_{x^{\left(k\right)}\subseteq x}{\left(-1\right)}^{|x^{\left(k\right)}|}I\left(x_{1}^{\left(k\right)};x_{2}^{\left(k\right)}\cdots ;x_{n_{k}}^{\left(k\right)}\right)$$

where $n_{k}$ is the dimension of the subset $x^{\left(k\right)}$.

Therefore, co-information can be used as a substitute to express mutual information based on Equation (7). Set theory has been used as an interpretation to explain the definition of co-information, i.e., $I\left(x_{1};x_{2};\cdots ;x_{n}\right)=f\left(X_{1}\cap X_{2}\cap \cdots \cap X_{n}\right)$ [44]. Equation (7) seems like a derivation of inclusion-exclusion principle.

If Equation (7) is substituted into Equation (3), the mutual information between multi-dimensional feature vector and label will be represented by co-information:

(8) $$I\left(x;y\right)=\sum_{i=1}^{n}I(x_{i};y)-\sum_{i=1}^{n-1}\sum_{j=i+1}^{n}I\left(x_{i};x_{j};y\right)+\cdots +{\left(-1\right)}^{n-1}I\left(x_{1};x_{2};\cdots ;x_{n};y\right)$$

#### 2.3.2. Feature Weight Estimation

The focus of this paper is heterogeneous bio-signal fusion for mental workload estimation. The calculation of the feature weights is indispensable for signal fusion. Because of the effectiveness of feature selection, mutual information method provides a promising application to calculating the dependency and redundancy information, which can extend to feature weight estimation.

Equation (8) gives the expanded form of mutual information which can be used to simplify the calculation by the truncated method:

(9) $$I\left(x;y\right)\approx \lambda \sum_{i=1}^{n}I\left(x_{i};y\right)-\sum_{i=1}^{n}\sum_{j=i+1}^{n-1}I(x_{i};x_{j};y)$$

Each component of Equation (9) has the particular significance. $I(x_{i};y)$ denotes the dependency information which interprets the relevance between the feature vector and label. Nevertheless, $I(x_{i};x_{j};y)$ denotes the redundancy information which should be limited. λ is a constant to adjust the ratio of dependency and redundancy information.

Because of the lower computational complexity, Equation (9) has been widely used to estimate the mutual information for feature selection. The goal of these feature selection methods is to find the optimal feature subset of x. If $x^{*}$ represents one of the subsets of x, the objective function is as follows:

(10) $$\max_{x^{*}\subseteq x}\left\{I(x^{*};y)\right\}=\max_{x^{*}\subseteq x}\left\{\lambda \sum_{i=1}^{n}I(x_{i}^{*};y)-\sum_{i=1}^{N-1}\sum_{j=i+1}^{N}I(x_{i}^{*};x_{j}^{*};y)\right\}$$

Though the objective Equation (10) represents the feature selection methods clearly, feature weight parameters can be supplemented to explain the formulation more clearly. If w denotes the feature weight vector of x, $w_{i}\in \left\{0,1\right\}$ can represent whether $x_{i}$ is included in the subset $x^{*}$. The Equation (10) will be transformed as:

(11) $$\max_{w_{i}\in \left\{0,1\right\}}\left\{\lambda \sum_{i=1}^{n}w_{i}I\left(x_{i};y\right)-\sum_{i=1}^{N-1}\sum_{j=i+1}^{N}w_{i}w_{j}I\left(x_{i};x_{j};y\right)\right\}$$

In the feature selection methods, $w_{i}$ is forced to be a boolean variable. However, if $w_{i}$ is limited as a positive real number from 0 to 1, it will be promising to optimize the feature wight based on Equation (11). Therefore, the original objective function for feature weight estimation proposed in this paper is as follows:

(12) $$\max_{w_{i}\in \left[0,1\right]}\left\{\lambda \sum_{i=1}^{n}w_{i}I\left(x_{i};y\right)-\sum_{i=1}^{N-1}\sum_{j=i+1}^{N}w_{i}w_{j}I\left(x_{i};x_{j};y\right)\right\}$$

Inspired by [44], this study uses mutual information matrix Q to simplify the objective function:

(13) $$Q=\left\{\begin{array}{ccc} I(x_{1};y) & \cdots & -I(x_{1};x_{n};y)\\ -I(x_{1};x_{2};y) & \cdots & -I(x_{2};x_{n};y)\\ \vdots & \ddots & \vdots \\ -I(x_{1};x_{n};y) & \cdots & I(x_{n};y) \end{array}\right\}$$

The mutual information matrix Q can be divided into the dependency matrix D and redundancy matrix R.

(14) $$D=\left\{\begin{array}{c} I(x_{1};y)\\ \vdots \\ I(x_{n};y) \end{array}\right\};R=\left\{\begin{array}{ccc} 0 & \cdots & -I(x_{1};x_{n};y)\\ -I(x_{1};x_{2};y) & \cdots & -I(x_{2};x_{n};y)\\ \vdots & \ddots & \vdots \\ -I(x_{1};x_{n};y) & \cdots & 0 \end{array}\right\}$$

Therefore, the objective function for feature weight estimation can be derived as Equation (15):

(15) $$\max_{w_{i}\in \left[0,1\right]}\left\{\lambda w^{T}D-w^{T}\mathrm{Rw}\right\}$$

Though the objective function of feature weight estimation has been proposed, it is hard to solve Equation (15) which is a non-convex problem and the solution may be over-fitting. As Equation (16) shows, one of the feasible options is to add a $\ell_{2}$ norm into the objective function.

(16) $$\max_{w_{i}\in \left[0,1\right]}\left\{\lambda w^{T}D-w^{T}\mathrm{Rw}-\gamma w^{T}w\right\}$$

The significance of adding $\ell_{2}$ norm $\gamma w^{T}w$ is threefold. First, it is a practical method to solve over-fitting problems. Second, $\ell_{2}$ norm can be used as a sparse item which can adjust the scale of w and prevent too many features having big weights. Third, considering that $w^{T}\mathrm{Rw}+\gamma w^{T}w\;=\;w^{T}\left(R+\gamma I\right)w$, if R+γI is a semi-definite matrix, Equation (16) will be a convex problem. Then, a suitable γ can convert the non-convex problem to the convex problem which is easier to be solved. Therefore, $\gamma \geq |\min\left\{\mathrm{eig}(R)\right\}|$ is an important precondition to solve Equation (16), where eig(R) represents the eigenvalues of R. The final objective function can be defined as Equation (17), which is named as interactive mutual information modeling (IMIM).

(17) $$\begin{array}{c} \underset{w}{\mathrm{maximize}}\;\;\lambda w^{T}D-w^{T}\left(R+\gamma I\right)w\\ \mathrm{subject}\;\mathrm{to}\;\;\;w_{i}\in \left[0,1\right]\;\mathrm{for}\;i=1,\cdots ,n\\ \;\;\;\;\;\;\;\;\;\;\;\;\;\;\;\;\;\;\;\gamma =|\min\left\{\mathrm{eig}(R)\right\}|\\ \;\;\;\;\;\;\;\;\;\;\;\;\;\;\;\;\;\;\;\lambda =\mathrm{constant} \end{array}$$

In Equation (17), the objective function consists of 2 parts. $w^{T}D$ represents the dependency information and $w^{T}\left(R+\gamma I\right)w$ represents the redundancy information. λ is a constant to adjust the ratio of the two parts. A big λ indicates the importance of increasing the dependency information. Conversely, the small one means that to eliminate the redundancy information will be better. In fact, λ needs to be chosen according to different conditions, and Section 3.3 will explain this procedure.

Equation (17) is a typical example of quadratic programming problems, which can be solved using convex optimization toolbox. Since R+γI is a semi-definite matrix, this equation can be solved by several methods with the computational cost of polynomial time. For example, primal interior point method can solve the problem with $O(n^{3}L)$, where *L* represents the input size [45]. To solve this convex quadratic programming problem, we use YALMIP interface with MOSEK solver which is free for academic use [46,47]. MOSEK is one of the most efficient solvers to optimize the linear, quadratic and conic problems. Though MOSEK solver may not have the best performance for solving Equation (17), it is designed to exploit sparsity to reduce storage usage and computational time [47]. MOSEK solver can be used to solve several thousand dimensional vectors in nearly 2 min with Intel(R) Core(TM) i5-3470 CPU. This study just utilized MOSEK solver to validate the proposed method.

#### 2.3.3. Heterogeneous Bio-Signal Fusion

This study extracted three kinds of feature vectors including EEG power spectral density (SPD) feature vector, EEG event-related potential (ERP) feature vector and ECG feature vector for heterogeneous bio-signal fusion. The normalization of the feature vectors is the precondition for further analysis. However, it is not the point of this paper. We just chose the unity-based normalization method according to Equation (18).

(18) $$x^{\prime }=\frac{x-\min(x)}{\max(x)-\min(x)}$$

ALL of the features will be brought into [0,1], which is convenient to adjust the importance of the features based on the feature weight vector. This paper subsequently develops feature level, and classifier level fusion methods based on IMIM for mental memory workload estimation.

##### Feature Level Fusion

Feature level fusion methods should combine the three feature vectors into a new feature vector and put it into one classifier. After the normalization of the feature vector, the feature weights can be calculated to adjust the scale of each feature by Equation (17). Given the feature vector $x=\{x_{1},x_{2},\cdots ,x_{n}\}$, the weighted feature vector could be represented by $x^{\prime }=\left\{w_{1}x_{1},w_{2}x_{2},\cdots ,w_{n}x_{n}\right\}$. Because of the sparsity of w, many elements of the feature vector $x^{\prime }$ should be 0. After removing the invalid features, we can obtain the fused feature vector $x^{\prime \prime }$. k-NN and SVM are used to validate the performance of the fused feature vector and develop the feature level fusion methods. This kind of methods is named as interactive mutual information modeling for feature level fusion (IMIM-F). It is noteworthy that IMIM-F is not suitable for quite a few classifiers which are not sensitive to feature weights, such as decision tree.

##### Decision Level Fusion

In decision level fusion methods, different feature vectors are put into the classifiers respectively. And the classification scores are combined to calculate the final results. In this study, k-NN and SVM are used to validate the performance of classifier level fusion methods for mental workload estimation.

In order to obtain the coefficients of the weighted average of classification scores, three feature weight vectors ($w^{\left(1\right)}$, $w^{\left(2\right)}$, $w^{\left(3\right)}$) are calculated based on Equation (17) using EEG PSD feature vector, EEG ERP feature vector and ECG feature vector. The weight $\beta_{i}$ of each classifier is defined as the optimized value in Equation (17). This method is named as interactive mutual information modeling for classifier level fusion (IMIM-C).

(19) $$\beta_{i}=w^{(i)T}D-\lambda w^{(i)T}\left(R+\gamma I\right)w^{\left(i\right)}$$

As Equation (19) shows, the calculation of $w^{\left(i\right)}$ is the precondition to obtain $\beta_{i}$. $w^{\left(i\right)}$ can adjust the dependency and redundancy information in each feature vector and it is helpful to strengthen the performance of each classifier. Therefore, different from the traditional classifier level fusion methods, IMIM-C changes the scale of each feature using $w^{\left(i\right)}$ before the classification. Each feature vector $\left\{x_{1}^{\left(i\right)},x_{2}^{\left(i\right)},\cdots ,x_{n_{i}}^{\left(i\right)}\right\}$ is transformed as $\left\{w_{1}^{\left(i\right)}x_{1}^{\left(i\right)},w_{2}^{\left(i\right)}x_{2}^{\left(i\right)},\cdots ,w_{n_{i}}^{\left(i\right)}x_{n_{i}}^{\left(i\right)}\right\}$, where i∈{1,2,3}. After that the 3 transformed feature vectors will be fed into the classifiers for classifier level fusion. The final predication is the weighted average of the classification scores based on $\beta_{i}$.

## 3. Results

This paper collects EEG, ECG signals to estimate mental workload based on heterogeneous signal fusion. Feature level fusion methods combine various types of features into a whole feature vector and adjust the weight of each feature for information fusion. Decision level fusion methods suppose that every feature vector is independent. They estimate the weight of each classifier trained by each feature vector. The final prediction is the weighted average of the classification scores. Experiment validates IMIM-F and IMIM-C based on the features which are discussed in Section 2.2.

Cross-validation is necessary to separate training, and test datasets when the amount of the data is limited. It is also indispensable to limit the over-fitting problems during the training process of the classifiers. Over-fitting is a phenomenon that the machine learning algorithm is so complicated that the internal details of the training dataset are over concerned. The classifier will be disabled to generalize new data. In this paper, a method named as “leave-one-proband-out” is used for cross-validation [48]. It uses the samples of one subject as the test dataset and the samples of the other nine subjects as the training dataset. This paper selects the test dataset from 10 subjects in order and repeats the classification process 10 times. There are two advantages of using this method. First, “leave-one-proband-out” operates similarly to 10 fold cross-validation and it does not use any test data in the training process. It can evaluate the generalization ability of the classifiers and limit over-fitting issues based on the prediction of the test dataset which can be assumed as unseen data. Second, it can ensure the effectiveness of the experiment for subject-independent application [48]. The average accuracy and standard deviation are obtained based on the 10 classification results.

### 3.1. Analysis of the N-Back Task

Physiological signals were recorded in 3 different mental memory workload levels based on the 1-, 2-, 3-back tasks. It is necessary to validate that the 1-, 2-, 3-back tasks had different difficulty and the subjects suffered from different workload. Table 2 describes the results of the NASA Task Load Index (NASA TLX). Table 3 shows the performance of the subjects during these tasks.

The NASA TLX is a subjective questionnaire to validate the effectiveness of the n-back task. Table 2 presents the self-rating results of all subjects. The results of the NASA TLX increase with the more-back tasks, with the average scores of 29.3 ± 6.4, 49.5 ± 4.5 and 69.6 ± 6.9 for the 1-, 2-, 3-back tasks respectively (repeated measures ANOVA: $F_{\left(2,27\right)}$ = 111.52, p<0.01). Tukey *post hoc* tests show that the subjects suffered from different mental workload during these tasks.

Besides the subjective measurement, Table 3 provides an objective validation of the mental workload tasks according to an inference that high mental workload will induce more errors and make people unresponsive. During the 1-back, 2-back, 3-back tasks, the response accuracy continues to decline (repeated measures ANOVA: $F_{\left(2,27\right)}$ = 23.9, p<0.01) and the reaction time is prolonged (repeated measures ANOVA: $F_{\left(2,27\right)}$ = 81.6, p<0.01). Tukey *post hoc* tests validate the differences of the three tasks. In the 1-back task, the average accuracy is 93.3%, and the standard deviation is 4.3%, which implies that all of the subjects accomplished this task successfully and they only suffered from the low memory workload. Nevertheless, the average accuracy decreases to 90.6% in the 2-back task, which indicates that the subjects needed to pay more attention and had the increased mental workload. The correct rate of the 3-back task is only 57.3% that is much lower than the previous two tasks. The subjects had the highest mental workload in the 3-back task since this task was too difficult. The time interval from the visual stimulus to the pressing of the keyboard was collected to analyze the reaction time of the subjects. The reaction time increases from the 1-back task to the 3-back task, which demonstrates that the 1-, 2-, 3-back tasks can induce low, medium and high mental memory workload respectively. Both the subjective and objective results confirm the differences of the 1-, 2-, 3-back tasks, which validates the inducing of three mental workload levels.

### 3.2. Data Recording

**Figure 3:**
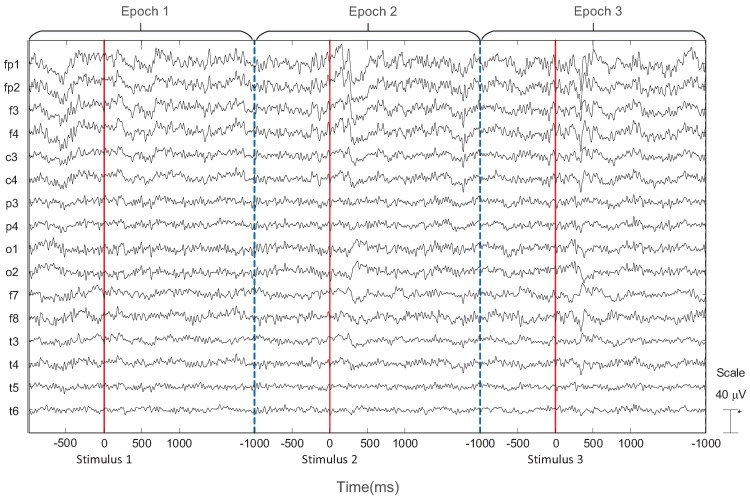
EEG signal collected from one subject after artifact removing. An example consists of 3 epochs during the 1-back task.

**Figure 4:**
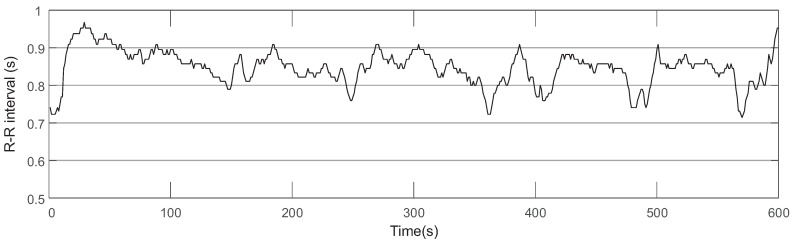
R-R intervals calculated based on the ECG signal which is collected from one subject during the 1-back task. All of the R-R intervals are longer than 0.5 s.

EEG and ECG signals were collected from ten subjects during the 1-, 2-, 3-back tasks to prove the effectiveness of IMIM for mental memory workload estimation. Figure 3 shows an example of the EEG signal which consists of 3 epochs collected from one subject during the 1-back task. This experiment used ADJUST algorithm to remove the artifacts of the EEG signal based on ICA [25]. ECG features were extracted based on R-R intervals provided by a patient monitor manufactured by Mindray company. Figure 4 shows an example of R-R intervals based on the ECG signal collected from one subject during the 1-back task.

As Section 2.2 shows, EEG power spectral density (PSD) features, EEG event-related potential (ERP) features and ECG features are extracted to validate the signal fusion methods for mental memory workload estimation. Usually, signal fusion methods consist of three categories: feature level fusion methods, classifier level fusion methods and the methods between feature level and classifier level fusion. The former two types of methods are the most popular for signal fusion, and this study validates IMIM in both feature level and classifier level fusion conditions.

### 3.3. Feature Level Fusion

Feature level fusion is an advanced strategy in comparison with classifier level fusion. It integrates all feature vectors and takes into account the relevance between any two features. Feature level fusion usually utilizes the information of the features more effectively and acquires the better performance than classifier level fusion.

#### 3.3.1. Parameter Adjustment for Feature Weight Estimation

As Section 2.3 shows, the parameter λ in Equation (17) is important to adjust the scale of each feature. A small λ will increase the consideration of redundancy information and tend to reduce the feature weights to eliminate the it. IMIM will estimate more feature weights as 0, and the fused feature vector will become sparse. The process of the determination of λ based on the single feature vectors and the fused feature vectors should be explained to explore the most effective feature weights for different feature vectors.

**Figure 5:**
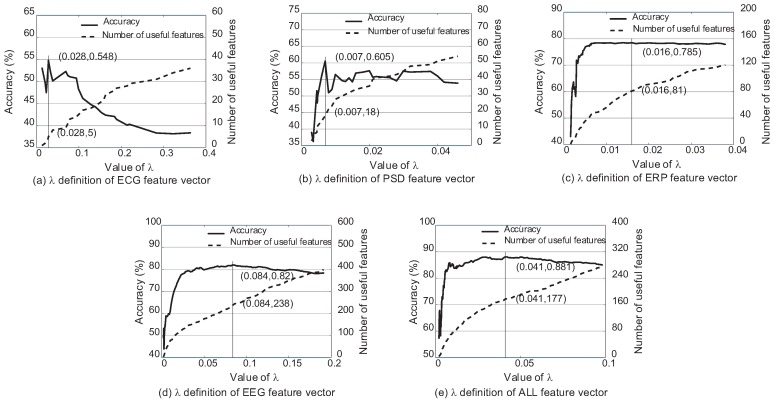
The classification accuracy using the single feature vectors (PSD feature vector, ERP feature vector, ECG feature vector) and fused vectors (EEG feature vector, ALL feature vector) according to λ based on k-NN (k = 3).

**Figure 6:**
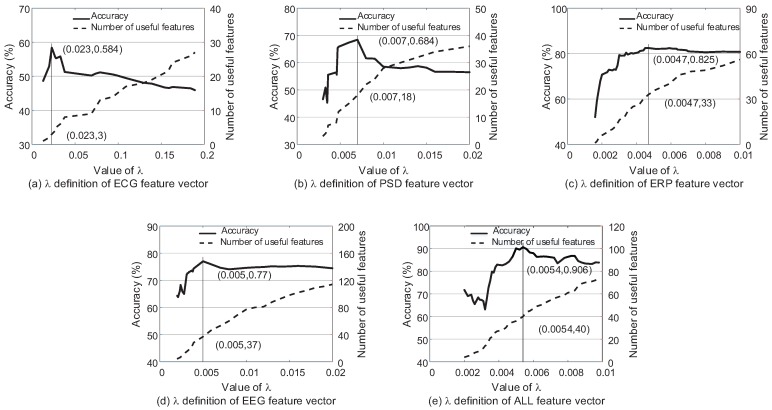
The classification accuracy using the single feature vectors (PSD feature vector, ERP feature vector, ECG feature vector) and fused vectors (EEG feature vector, ALL feature vector) according to λ based on SVM (C = 10${}^{-3}$).

This paper presents the selection process of λ based on k-NN (k = 3) and soft margin SVM (C = $10^{-3}$) classifiers. The classification performance and the number of features whose weights are bigger than 0 according to λ are shown as Figure 5 and Figure 6. There are several preliminaries of the features to describe Figure 5 and Figure 6 clearly. ECG features consist of heart rate, R-R interval and different definitions of heart rate variability according to HRVAS [39]. PSD features and ERP features are extracted from EEG according to the power spectral density and moments of the stimuli respectively. EEG feature vector is the combination of the PSD features and ERP features. ALL feature vector is the combination of EEG features and ECG features. Among different feature vectors involved in this experiment, EEG feature vector and ALL feature vector are both the fused feature vectors. PSD feature vector, ERP feature vector and ECG feature vector are three single feature vectors.

Figure 5 and Figure 6 explain the selection process of λ using different feature vectors according to k-NN (k = 3) and SVM (C = 10${}^{-3}$) respectively. For k-NN, it reaches the highest classification accuracy when λ=0.028,0.007,0.016,0.084,0.041 according to ECG feature vector, EEG PSD feature vector, EEG ERP feature vector, EEG feature vector and ALL feature vector respectively. Different from k-NN, the most suitable λ=0.023,0.007,0.0047,0.005,0.0054 for SVM. It is obvious that the appropriate λ is different for different classifiers and feature vectors. Each feature vector has the exact amount of dependency and redundancy information. Different classifiers also have different ability to utilize the information. The parameter λ is exactly the tool to adjust the ratio of the two kinds of information. Therefore, λ cannot be set as a stable constant, and it should be re-selected in each model.

Figure 5 and Figure 6 also explain the changes in classification accuracy with the increase in the number of the useful features whose weights are bigger than 0. The trend is the same for all curves that the accuracy increases first and then drops because of the increase of redundancy information with the greater feature weight vector w.

#### 3.3.2. Necessity of Signal Fusion and Parameter Selection of the Classifiers

**Figure 7:**
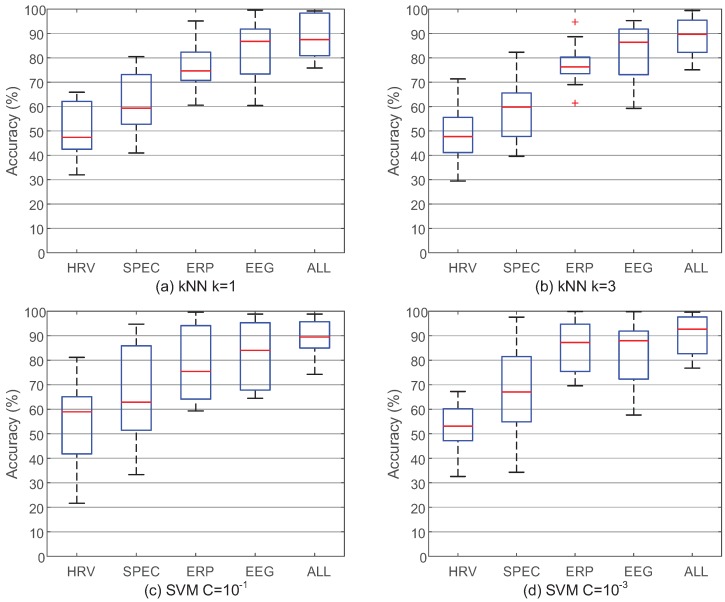
The performance of IMIM-F between the single feature vectors and the fused feature vectors based on different classifiers. k-NN and SVM classifiers are chosen to validate the necessity of signal fusion.

It is reasonable that better classification accuracy will be reached based on the fused feature vector which can provide more information than a single feature vector. Nevertheless, there are still two problems which may reject this inference. First, if the feature level fusion method is not appropriate and many redundant features are mistakenly considered important, the performance of the fused feature vector will be even worse than the single feature vectors. Second, if the classification accuracy based on the single feature vector is high enough, there will be no need to develop signal fusion framework. Therefore, Figure 7 compares the classification results between the single and fused feature vectors based on IMIM-F to validate the usefulness of this method and to explain the necessity of signal fusion.

Figure 7 compares the single and fused feature vectors based on different classifiers. EEG PSD feature vector and EEG ERP feature vector have better performance than ECG feature vector, which emphasizes the importance of EEG features to estimate mental workload. EEG feature vector is the combination of EEG PSD feature vector and EEG ERP feature vector. The classification accuracy of EEG feature vector is obviously better than PSD feature vector and ERP feature vector, which validates the effectiveness of IMIM-F for homogeneous feature level fusion. ALL feature vector consists of 3 single feature vectors based on IMIM-F, and the performance is improved significantly. Therefore, the best performance based on a single feature vector is not sufficient, and it is still necessary to fuse different feature vectors. The fused feature vectors reach the higher classification accuracy, which proves the effectiveness of the proposed method. The classification results in Figure 7 can answer the two questions raised in the preceding paragraph.

Figure 7 also presents the parameter selection for k-NN (k = 1, 3) and soft margin SVM (C = 10${}^{-1}$, 10${}^{-3}$) classifiers. The k-NN classifier reaches better accuracy when k = 3. The parameter C affects the performance of SVM obviously, and it reaches higher accuracy when C = 10${}^{-3}$.

Table 4 presents the classification results using ALL feature vector based on different parameters. The parameter k of the k-NN classifier is chosen from (1, 3, 5, 10). It is most suitable to use k = 3 for k-NN, which achieves the accuracy of 88.1%. The parameter C of soft margin SVM is chosen from (C = 10${}^{-1}$, 10${}^{-2}$, 10${}^{-3}$, 10${}^{-4}$) and it is evident that SVM reaches the highest classification accuracy of 90.6% when C = 10${}^{-3}$. Therefore, the optimum parameters are k = 3 and C = 10${}^{-3}$ for k-NN and SVM respectively.

#### 3.3.3. Comparison of Feature Level Fusion Methods

**Figure 8:**
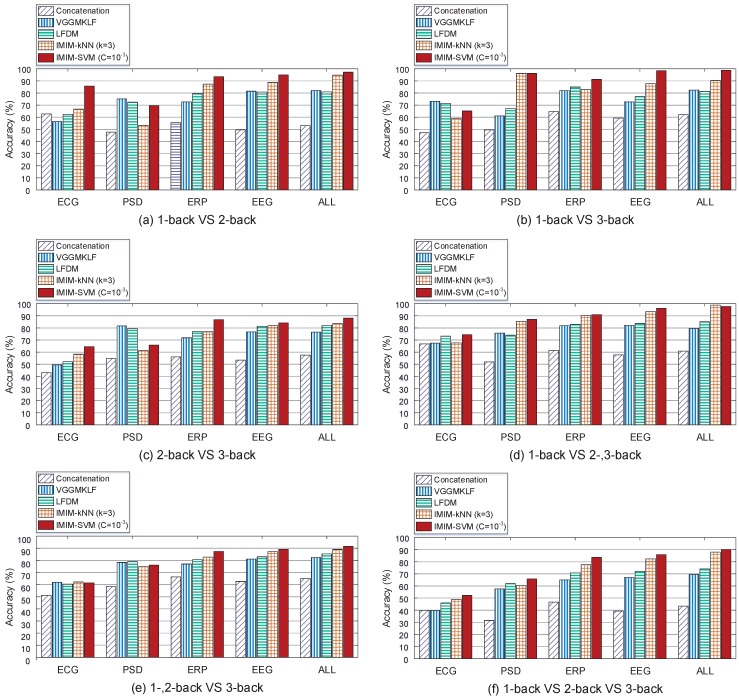
Classification accuracy based on Concatenation method, VGGMKL, LFDM and IMIM-F for feature level fusion. Different single feature vectors and fused feature vectors are all used for mental workload estimation. 6 pairs of mental workload conditions (1-back VS 2-back, 1-back VS 3-back, 2-back VS 3-back, 1 and 2-back VS 3-back, 1-back VS 2 and 3-back, 1-back VS 2-back VS 3-back) are used to compare the performance of the methods.

Recent researchers have proposed several feature level fusion methods to improve the classification accuracy based on the utilization of multi-modal signals recorded from different types of sensors. Concatenation, Multi-kernel learning, and linear dependency modeling based on probability have all been used to explore the feature level fusion problem. Figure 8 compares the proposed method IMIM-F (interactive mutual information modeling for feature level fusion) with Concatenation method, VGGMKL [49], and LFDM (linear feature dependency modeling) [50] to verify the advancement of IMIM.

Figure 8 compares the feature level fusion methods in 2 aspects. First, it compares the improvement of these methods from the single feature vectors to the fused feature vectors. Second, it investigates the ability of the methods to distinguish different mental workload conditions across 1-, 2-, 3-back tasks. Figure 8 shows the performance of the methods in 6 conditions (1-back VS 2-back, 1-back VS 3-back, 2-back VS 3-back, 1-back VS 2-, 3-back, 1-, 2-back VS 3-back, and 1-back VS 2-back VS 3-back). All of the methods have poor performance in 2-back VS 3-back, and 1-, 2-back VS 3-back conditions. It implies that the mental workload levels in 2-back task and 3-back task are similar. However, these methods reach higher accuracy in 1-back VS 2-back, 1-back VS 3-back and 1-back VS 2-, 3-back tasks, which represents the big differences between 1-back and more-back tasks. Figure 8 (f) measures the performance of different methods in 1-back VS 2-back VS 3-back condition, and it provides the most comprehensive evaluation. In this Figure, IMIM-F based on k-NN does not perform better than other methods using EEG PSD feature vector. However, IMIM-F methods outperform the others using all of the rest feature vectors. In the comparison of heterogeneous feature level fusion in 1-back VS 2-back VS 3-back condition, the classification accuracy of IMIM-F methods based on k-NN and SVM reach 88.0% and 90.6% respectively, which validates the advancement of IMIM-F in feature level fusion.

**Figure 9:**
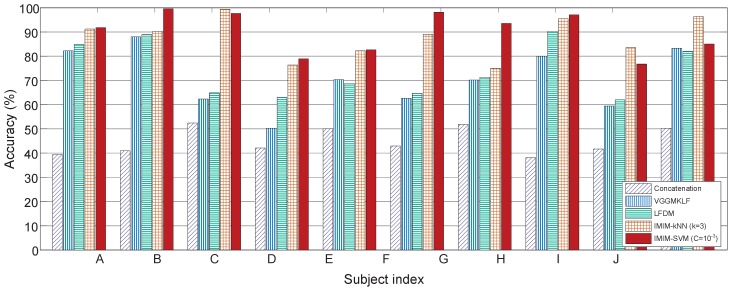
Comparison of feature level fusion methods according to ten subjects involved in this experiment. The subjects are numbered from A to J randomly.

This paper uses “leave-one-proband-out” strategy for cross-validation. This method not only limits over-fitting problems but also develops a subject-independent mental workload classification application. This strategy uses the data from one subject as the test dataset and the data from the other nine subjects as the training dataset. Figure 9 shows the classification accuracy in 1-back VS 2-back VS 3-back condition based on ALL feature vector according to ten subjects involved in this experiment.

Figure 9 shows that the traditional Concatenation method has the worst performance. Simply concatenating different feature vectors cannot improve the classification accuracy significantly. IMIM-F methods, including IMIM-F-kNN and IMIM-F-SVM, improve the classification accuracy significantly compared with Concatenation, LFDM, and VGGMKL (paired *t*-test, p<0.01). However, the difference between IMIM-F-kNN and IMIM-F-SVM is not significant (paired *t*-test, p=0.4306). IMIM-F outperforms the other methods based on k-NN (k = 3) and SVM (C = 10${}^{-3}$) for every subject, which validates that the over-fitting problems are limited, and IMIM-F is advanced for subject-independent mental workload estimation.

### 3.4. Decision Level Fusion

Decision level fusion is designed to combine the scores of different classifiers to improve the classification accuracy. The focus of these methods is to estimate the weight of each classifier and calculate the weighted average results. We can suppose that EEG power spectral density (PSD) feature vector, EEG event-related potential (ERP) feature vector, and ECG feature vector are independent. Decision level fusion methods usually put the three feature vectors into three classifiers respectively. For comparison, different classifier level fusion methods, including Average method, VGGMKL, LCDM (linear classifier dependency modeling), and IMIM-C (interactive mutual information modeling for classifier level fusion) are utilized to estimate mental memory workload.

#### 3.4.1. Decision Level Fusion Method Based on IMIM

Compared with traditional classifier level fusion methods, IMIM-C has a different characteristic. As Equation (19) shows, IMIM-C obtains the weight of each feature $w_{j}^{\left(i\right)}$ and the weight of each classifier $\beta_{i}$ simultaneously. Therefore, it can optimize the scale of each feature before the classification process to improve the performance.

**Figure 10:**
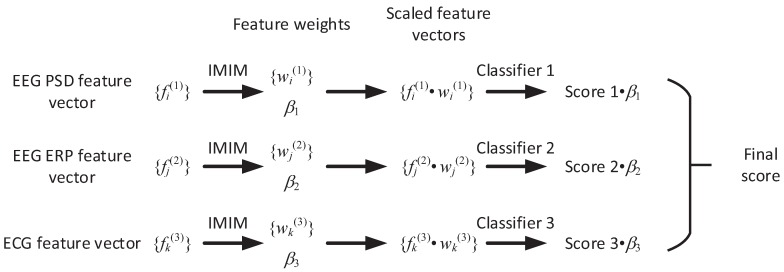
The framework of decision level fusion based on IMIM.

As Figure 10 shows, IMIM-C consists of four steps. First, this method calculates the weight of each feature $w_{p}^{\left(q\right)}$ and the weight of each feature vector $\beta_{q}$ based on IMIM. Second, this method calculates the scaled feature vector to adjust the weights of the features in each feature vector. Third, IMIM-C puts three different feature vectors into the classifiers and obtains 3 predictions. Fourth, the final decision is the weighted average of the classification results based on the feature vector weight $\beta_{q}$.

#### 3.4.2. Comparison of Different Classifier Level Fusion Methods

Section 3.3 has described the selection of parameter λ in Equation (17) based on each feature vector. As Equation (19) shows IMIM-C estimates the weights of the features and the feature vectors simultaneously. Table 5 displays the comparison between IMIM-C and other classifier level fusion methods based on k-NN (k=1, 3) and SVM (C = 10${}^{-1}$, 10${}^{-3}$) classifiers.

Researchers have proposed different kinds of classifier level fusion methods. The Average method uses the average score of several classifiers to obtain the final prediction. Multi-kernel learning (MKL) estimates the weights of different kernels based on several feature vectors and uses the weighted average kernel to train SVM for classifier level fusion, such as VGGMKL. Boost methods think of each classifier based on the single feature vector as a weak classifier and combine several weak classifiers for the better performance. LCDM improves the objective function of LP-B which is a multi-class variant of LPBoost [51]. Table 5 compares the proposed method IMIM-C with the Average method, VGGMKL, and LCDM.

SVM and k-NN are used to compare different classifier level fusion methods. The parameters of the classifiers are changed to demonstrate the effectiveness of IMIM-C and obtain better results. For k-NN, the number of the nearest neighbors is selected from {1, 3}. In the soft margin SVM, parameter C is chosen from {10${}^{-3}$, 10${}^{-1}$} and the classification accuracy will no longer change when C is larger than 10${}^{-1}$.

Table 5 presents the average accuracy and the standard deviation based on the cross-validation strategy named “leave-one-proband-out”. The results in 1-back VS 2-back VS 3-back condition provide the comprehensive analysis. In this condition, IMIM-C outperforms the other classifier level fusion methods for both SVM and k-NN classifiers, which indicates that IMIM-C is advanced for heterogeneous bio-signal fusion. IMIM-C reaches the best performance based on SVM when C = 10${}^{-3}$. It is evident that IMIM-C is sensitive to the parameter C and the small C is more suitable to avoid over-fitting. For k-NN, IMIM-C has the better performance when k is larger because it can utilize more information in the classification process.

Table 5 also presents the ability of the classifier level fusion methods to distinguish the mental workload levels according to six pairs of 1-, 2-, 3-back tasks. IMIM-C outperforms the other methods in each condition based on each classifier, which validates the advancement of IMIM-C. Similar to the results of feature level fusion, all of the methods reach the higher accuracy in 1-back VS 2-back, 1-back VS 3-back and 1-back VS 2-, 3-back conditions. The mental workload induced by 2-, 3-back tasks is obviously higher than 1-back task. However, it is sometimes difficult to distinguish the mental workload levels in 2-back and 3-back tasks. The results indicate that the features extracted from EEG and ECG signals are different between the lowest workload and the increased workload conditions. But it is challenging to distinguish various levels of high mental workload.

**Figure 11:**
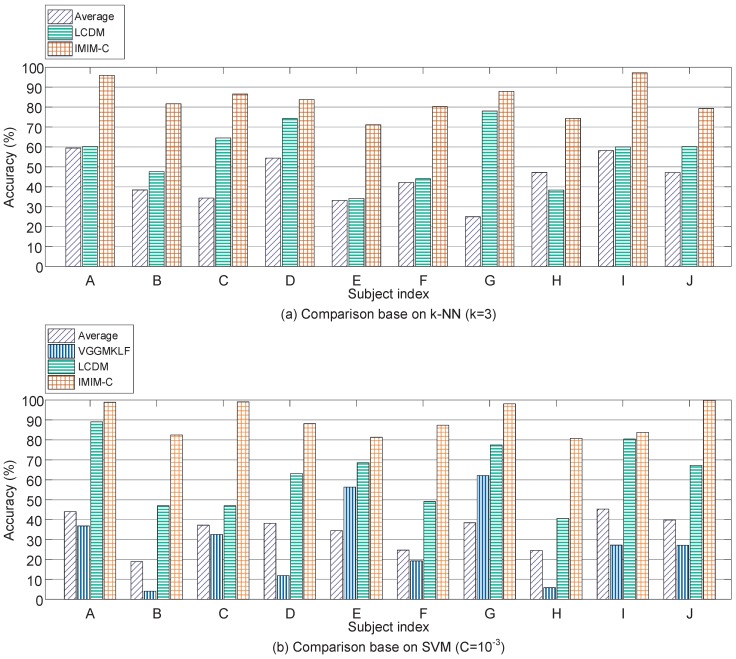
Comparison of classifier level fusion methods according to 10 subjects involved in this experiment. The subjects are numbered from A to J randomly.

Figure 11 shows the classification accuracy for each subject based on “leave-one-proband-out” cross-validation method. IMIM-C outperforms the other methods based on k-NN (k = 3) and SVM (C = 10${}^{-3}$) (paired *t*-test, p<0.01), which demonstrates that over-fitting problems are limited in the proposed method. IMIM-C is promising for subject-independent mental workload estimation.

### 3.5. Comparison with Other Research for Mental Workload Estimation

Section 3.3 and Section 3.4 have described the results of feature level fusion and classifier level fusion. However, the methods used for comparison in the former sections are usually designed for various applications. Table 6 compares IMIM with the other methods developed mainly for mental workload estimation.

EEG is indispensable for mental workload estimation, and some researchers focused on this problem only using EEG signal [31,37,52]. Simultaneously, the fusion of EEG, ECG, and EOG has been explored to improve the classification accuracy. Some researchers concatenated features from heterogeneous signals [28,30]. However, the accuracy was not increased obviously because of the redundant features. Feature selection method was used before signal fusion to eliminate the unnecessary features and improve the performance of the classifiers [17]. To utilize the information of the heterogeneous feature vectors, we estimate the feature weights based on interactive mutual information modeling (IMIM). IMIM-F reaches the highest precision in Table 6 at 91% and IMIM-C also outperforms the other methods. IMIM improves the estimation of feature weights and develops the feature-level, classifier level fusion methods. It maximizes the effective information contained in various feature vectors and improves the performance based on different classifiers.

### 3.6. Discussion

The physiological signals recorded during 1-, 2-, 3-back tasks are used to validate IMIM for mental workload estimation. The former sections have presented the classification results and comparison. However, there are still some critical issues.

In the comparison of feature level fusion methods, IMIM-F reaches the highest precision compared with Concatenation method, LFDM, and VGGMKL. The proposed method has better performance using the fused feature vector than a single feature vector. However, there is no noticeable improvement based on the other methods when they combine heterogeneous signals. It indicates the difficulty in utilizing the dependency information and eliminating the redundancy information simultaneously. IMIM-F estimates the redundancy information based on the optimization of mutual information and combines the features based on the feature weights to utilize the dependency information. However, the other methods do not consider the interaction between any two features in the whole feature vector correctly. The results of feature level fusion validate the advancement of IMIM-F.

Classifier level fusion methods focus on the estimation of classifier weights. The performance of classifier level fusion is usually worse than feature level fusion because feature level fusion considers the interaction of all features and utilizes more information. Therefore, besides the IMIM method, all the signal fusion methods used in this study have a worse performance in classifier level fusion than feature level fusion. IMIM improves the performance in classifier level fusion significantly because of the optimization of each feature vector. Equation (17) estimates the weight of each feature ${w_{p}}^{\left(q\right)}$ and the weight of each feature vector $\beta_{q}$ simultaneously. The redundancy information and dependency information are taken into account in one feature vector based on ${w_{p}}^{\left(q\right)}$. Therefore, IMIM-C has refined each feature vector before classification, which can improve the classification accuracy evidently.

This paper also compares IMIM with other research for mental workload estimation. EEG is the most important signal for mental workload estimation, and many research groups proposed methods only based on EEG. In recent years, multi-modal biometric signal fusion has been one of the focuses, and the feature combination of EEG, ECG, EOG increases the mental workload classification accuracy. However, these researchers just concatenated different feature vectors, which may induce the redundancy information. As Table 6 shows, compared with other mental workload estimation methods, IMIM has the best performance. The results imply that heterogeneous bio-signal fusion is promising to classify different mental workload states. IMIM is an advanced method in both feature level and classifier level fusion.

## 4. Conclusions

This paper focuses on mental workload estimation based on heterogeneous bio-signal fusion. The high mental workload is harmful to human health and may cause particular people, such as pilots, soldiers, crew, and surgeons, to commit serious mistakes. Though EEG is the primary physiological signal to reflect workload, additional information of other physiological signals, such as ECG, can be used to improve the performance of the classifiers. This paper improves the objective function of mutual information and utilizes the $\ell_{2}$ norm to transform the non-convex problem into the convex problem. Then interactive mutual information modeling (IMIM) is proposed to improve the mental workload estimation based on heterogeneous bio-signal fusion. IMIM extends the application of mutual information to estimating the weights of features and feature vectors for signal fusion. N-back task is utilized to induce different mental workload states. The proposed method fuses EEG power spectral density (PSD) features, EEG event-related potential (ERP) features and ECG features based on the data collected in 1-, 2-, 3-back tasks. The discussion and evaluation are conducted as threefold. First, IMIM-F (IMIM for feature level fusion) is compared with other feature level fusion methods. Second, IMIM-C (IMIM for classifier level fusion) is designed to compare with classifier level fusion methods, and it outperforms the other methods based on different classifiers. Third, it is necessary to compare IMIM with the previous research for mental workload estimation. Compared with recent study, IMIM reaches the highest classification accuracy because it fuses heterogeneous feature vectors based on the consideration of redundancy and dependency information. IMIM effectively improves the classification accuracy, and can be applied to monitoring mental workload. IMIM is also helpful to develop body sensor networks based on multi-modal physiological sensors. This study tries to propose the feature-engineering method which can apply to different classifiers only based on the n-back task. The future work is to develop modern machine learning algorithms cross different mental workload tasks.

## Figures and Tables

**Table 1:** Experiment with 3 sessions according to 1-, 2-, 3-back tasks.

	Step 1	Step 2	Step 3
Session 1	Rest (5 min)	200 trails of 1-back task (10 min)	NASA TLX (2 min)
Session 2	Rest (3 min)	200 trails of 2-back task (10 min)	NASA TLX (2 min)
Session 3	Rest (3 min)	200 trails of 3-back task (10 min)	NASA TLX (2 min)

**Table 2:** The scores of the NASA Task Load Index collected from the subjects involved in the 1-, 2-, 3-back tasks. The subjects are numbered from A to J.

Subject Index	A	B	C	D	E	F	G	H	I	J
1-back task	22	21	31	35	38	20	31	29	30	39
2-back task	44	55	46	51	43	52	45	53	52	54
3-back task	69	62	65	78	63	77	71	80	61	70

**Table 3:** Average performance including the response accuracy and the reaction time of the ten subjects during the 1-, 2-, 3-back tasks.

	1-Back Task	2-Back Task	3-Back Task
Response accuracy (%)	93.3 ± 4.5	90.6 ± 3.4	57.3 ± 11.6
Reaction time (ms)	535 ± 58	712 ± 127	960 ± 212

**Table 4:** Classification accuracy of k-NN (k = 1, 3, 10, 20) and SVM (C = 10${}^{-1}$, 10${}^{-2}$, 10${}^{-3}$, 10${}^{-4}$) using ALL feature vector based on IMIM.

	k-NN				SVM			
	k = 1	k = 3	k = 5	k = 10	C = 10${}^{-1}$	C = 10${}^{-2}$	C = 10${}^{-3}$	C = 10${}^{-4}$
Accuracy (%)	87.9	88.1	87.5	87.2	88.4	89.2	90.6	88.8
Standard Deviation (%)	8.5	7.9	8.7	8.3	9.4	9.6	8.1	7.8

**Table 5:** Comparison of the classifier level fusion methods based on SVM (C = 10${}^{-1}$, C = 10${}^{-3}$) and k-NN (k = 1, k = 3) for mental workload estimation. 6 pairs of mental workload conditions (1-back VS 2-back, 1-back VS 3-back, 2-back VS 3-back, 1 and 2-back VS 3-back, 1-back VS 2 and 3-back, 1-back VS 2-back VS 3-back) are used to compare the ability of the methods to classify the mental workload levels in 1-, 2-, 3-back tasks. VGG-MKL is a multi-kernel learning algorithm based on SVM.

Conditions	Methods	k-NN (%)		SVM (%)	
		k = 1	k = 3	C = 10${}^{-1}$	C = 10${}^{-3}$
1-back VS. 2-back	Average	53.0 ± 13.2	54.0 ± 12.6	54.0 ± 25.7	56.1 ± 29.1
	VGG-MKL	−	−	56.0 ± 17.4	55.3 ± 20.1
	LCDM	75.5 ± 19.0	75.4 ± 19.5	79.2 ± 17.0	81.1 ± 18.4
	IMIM-C	85.0 ± 7.7	87.7 ± 7.9	91.9 ± 11.6	97.1 ± 3.0
1-back VS. 3-back	Average	62.1 ± 14.7	61.9 ± 15.7	47.8 ± 26.0	49.1 ± 23.6
	VGG-MKL	−	−	45.8 ± 22.5	43.8 ± 21.4
	LCDM	66.1 ± 18.1	66.3 ± 14.1	78.1 ± 18.5	78.5 ± 18.1
	IMIM-C	89.8 ± 10.6	90.4 ± 9.8	96.4 ± 7.0	99.6 ± 0.9
2-back VS. 3-back	Average	57.5 ± 12.2	59.1 ± 13.9	48.6 ± 14.3	48.3 ± 12.5
	VGG-MKL	−	−	45.7 ± 28.3	44.2 ± 26.0
	LCDM	64.1 ± 18.5	70.4 ± 18.9	67.4 ± 12.3	66.9 ± 12.2
	IMIM-C	77.6 ± 9.8	79.2 ± 8.9	72.6 ± 39.1	86.6 ± 13.2
1-back VS. 2-back, 3-back	Average	60.8 ± 5.0	60.6 ± 4.3	64.1 ± 19.0	65.6 ± 18.4
	VGG-MKL	−	−	55.6 ± 21.1	53.9 ± 21.1
	LCDM	71.2 ± 14.5	70.3 ± 13.5	78.6 ± 16.1	79.9 ± 18.7
	IMIM-C	90.1 ± 5.4	90.9 ± 4.9	98.0 ± 3.8	98.8 ± 1.9
1-back, 2-back VS. 3-back	Average	65.0 ± 10.9	65.2 ± 11.6	47.8 ± 24.0	49.2 ± 21.9
	VGG-MKL	−	−	48.7 ± 14.5	48.1 ± 12.9
	LCDM	64.6 ± 10.9	70.9 ± 18.0	70.6 ± 11.8	71.8 ± 9.8
	IMIM-C	81.8 ± 9.6	83.1 ± 8.8	82.0 ± 36.3	91.1 ± 8.8
**1-back VS. 2-back VS. 3-back**	Average	43.4 ± 3.1	43.6 ± 3.9	34.3 ± 23.8	35.4 ± 22.5
	VGG-MKL	−	−	28.3 ± 18.6	26.3 ± 19.2
	LCDM	53.0 ± 2.5	56.2 ± 2.7	61.0 ± 3.3	63.0 ± 3.5
	IMIM-C	76.6 ± 10.8	78.5 ± 9.6	80.2 ± 25.4	89.9 ± 8.6

**Table 6:** Comparison of different mental workload estimation methods in recent years.

	Year	Signal	Classifier	Accuracy (%)
Christensen et al. [28]	2012	EEG, EOG, ECG	ANN	86
Brouwer et al. [37]	2012	EEG	SVM	84
Mühl et al. [31]	2014	EEG	LDA	80
Yin et al. [17]	2014	EEG, ECG	SVM	85
Estepp et al. [30]	2015	EEG, EOG, ECG	SVM	87
Zarjam et al. [52]	2015	EEG	ANN	86
This Study IMIM-F	2017	EEG, ECG	SVM	91
This Study IMIM-C	2017	EEG, ECG	SVM	90

## References

[1] Dai Y., Wang X., Li X., Tan Y. (2015). Sparse EEG compressive sensing for web-enabled person identification. Measurement.

[2] Baldwin C.L., Penaranda B.N. (2012). Adaptive training using an artificial neural network and EEG metrics for within- and cross-task workload classification. Neuroimage.

[3] Qian D., Wang B., Qing X., Zhang T., Zhang Y., Wang X., Nakamura M. (2017). Drowsiness Detection by Bayesian-Copula Discriminant Classifier Based on EEG Signals During Daytime Short Nap. IEEE Trans. Biomed. Eng..

[4] Roy R.N., Charbonnier S., Campagne A., Bonnet S. (2016). Efficient mental workload estimation using task-independent EEG features. J. Neural Eng..

[5] Chai R., Naik G.R., Nguyen T.N., Ling S.H., Tran Y., Craig A., Nguyen H.T. (2017). Driver Fatigue Classification With Independent Component by Entropy Rate Bound Minimization Analysis in an EEG-Based System. IEEE J. Biomed. Health Inform..

[6] Yin Z., Zhang J. (2017). Cross-session classification of mental workload levels using EEG and an adaptive deep learning model. Biomed. Signal Process. Control.

[7] Hefron R.G., Borghetti B.J., Christensen J.C., Kabban C.M.S. (2017). Deep long short-term memory structures model temporal dependencies improving cognitive workload estimation. Pattern Recognit. Lett..

[8] Hoover A., Singh A., Fishel-Brown S., Muth E. (2012). Real-time detection of workload changes using heart rate variability. Biomed. Signal Process. Control.

[9] Laurent F., Valderrama M., Besserve M., Guillard M., Lachaux J.P., Martinerie J., Florence G. (2013). Multimodal information improves the rapid detection of mental fatigue. Biomed. Signal Process. Control.

[10] Park S., Won M.J., Lee E.C., Mun S., Park M.C., Whang M. (2015). Evaluation of 3D cognitive fatigue using heart–brain synchronization. Int. J. Psychophysiol..

[11] Jagannath M., Balasubramanian V. (2014). Assessment of early onset of driver fatigue using multimodal fatigue measures in a static simulator. Appl. Ergon..

[12] Gergelyfi M., Jacob B., Olivier E., Zénon A. (2015). Dissociation between mental fatigue and motivational state during prolonged mental activity. Front. Behav. Neurosci..

[13] Wang X., Wang S., Ma J.J. (2007). An improved co-evolutionary particle swarm optimization for wireless sensor networks with dynamic deployment. Sensors.

[14] Verma G.K., Tiwary U.S. (2014). Multimodal fusion framework: A multiresolution approach for emotion classification and recognition from physiological signals. Neuroimage.

[15] Gravina R., Alinia P., Ghasemzadeh H., Fortino G. (2017). Multi-sensor fusion in body sensor networks: State-of-the-art and research challenges. Inf. Fusion.

[16] Hogervorst M.A., Brouwer A.M., van Erp J.B.F. (2014). Combining and comparing EEG, peripheral physiology and eye-related measures for the assessment of mental workload. Front. Neurosci..

[17] Yin Z., Zhang J. (2014). Operator functional state classification using least-square support vector machine based recursive feature elimination technique. Comput. Meth. Programs Biomed..

[18] Aydın S., Tunga M.A., Yetkin S. (2015). Mutual information analysis of sleep eeg in detecting psycho-physiological insomnia. J. Med. Syst..

[19] Scharinger C., Soutschek A., Schubert T., Gerjets P. (2015). When flanker meets the n-back: What EEG and pupil dilation data reveal about the interplay between the two central-executive working memory functions inhibition and updating. Psychophysiology.

[20] Kirchner W.K. (1958). Age differences in short-term retention of rapidly changing information. J. Exp. Psychol..

[21] Antonenko P., Paas F., Grabner R., van Gog T. (2010). Using Electroencephalography to Measure Cognitive Load. Educ. Psychol. Rev..

[22] Wang S., Gwizdka J., Chaovalitwongse W.A. (2016). Using Wireless EEG Signals to Assess Memory Workload in the *n*-Back Task. IEEE Trans. Hum.-Mach. Syst..

[23] Al-Qazzaz N., Hamid Bin Mohd Ali S., Ahmad S., Islam M., Escudero J. (2017). Automatic Artifact Removal in EEG of Normal and Demented Individuals Using ICA–WT during Working Memory Tasks. Sensors.

[24] Delorme A., Makeig S. (2004). EEGLAB: An open source toolbox for analysis of single-trial EEG dynamics including independent component analysis. J. Neurosci. Methods.

[25] Mognon A., Jovicich J., Bruzzone L., Buiatti M. (2011). ADJUST: An automatic EEG artifact detector based on the joint use of spatial and temporal features. Psychophysiology.

[26] Ke Y., Qi H., Zhang L., Chen S., Jiao X., Zhou P., Zhao X., Wan B., Ming D. (2015). Towards an effective cross-task mental workload recognition model using electroencephalography based on feature selection and support vector machine regression. Int. J. Psychophysiol..

[27] Sauvet F., Bougard C., Coroenne M., Lely L., Van Beers P., Elbaz M., Guillard M., Leger D., Chennaoui M. (2014). In-Flight Automatic Detection of Vigilance States Using a Single EEG Channel. IEEE Trans. Biomed. Eng..

[28] Christensen J.C., Estepp J.R., Wilson G.F., Russell C.A. (2012). The effects of day-to-day variability of physiological data on operator functional state classification. Neuroimage.

[29] Ke Y., Qi H., He F., Liu S., Zhao X., Zhou P., Zhang L., Ming D. (2014). An EEG-based mental workload estimator trained on working memory task can work well under simulated multi-attribute task. Front. Hum. Neurosci..

[30] Estepp J.R., Christensen J.C. (2015). Electrode replacement does not affect classification accuracy in dual-session use of a passive brain-computer interface for assessing cognitive workload. Front. Neurosci..

[31] MÃ Hl C., Jeunet C., Lotte F. (2014). EEG-based workload estimation across affective contexts. Front. Neurosci..

[32] Casson A.J. (2014). Artificial Neural Network classification of operator workload with an assessment of time variation and noise-enhancement to increase performance. Front. Neurosci..

[33] Wang Z., Hope R.M., Wang Z., Ji Q., Gray W.D. (2012). Cross-subject workload classification with a hierarchical Bayes model. Neuroimage.

[34] Zhao Y.X., Chou C.H. (2016). Feature Selection Method Based on Neighborhood Relationships: Applications in EEG Signal Identification and Chinese Character Recognition. Sensors.

[35] Myrden A., Chau T. (2015). Effects of user mental state on EEG-BCI performance. Front. Hum. Neurosci..

[36] Yin Z., Zhang J. (2014). Identification of temporal variations in mental workload using locally-linear- embedding-based EEG feature reduction and support-vector-machine-based clustering and classification techniques. Comput. Meth. Programs Biomed..

[37] Brouwer A.M., Hogervorst M.A., van Erp J.B., Heffelaar T., Zimmerman P.H. (2012). Estimating workload using EEG spectral power and ERPs in the n-back task. J. Neural Eng..

[38] Dyke F.B., Leiker A.M., Grand K.F., Godwin M.M., Thompson A.G., Rietschel J.C., McDonald C.G., Miller M.W. (2015). The efficacy of auditory probes in indexing cognitive workload is dependent on stimulus complexity. Int. J. Psychophysiol..

[39] Ramshur J. (2010). Design, Evaluation, and Application of Heart Rate Variability Analysis Software (HRVAS). Master’s Thesis.

[40] Zhang P., Wang X., Li X., Dai P. EEG feature selection based on weighted-normalized mutual information for mental fatigue classification. Proceedings of the 2016 IEEE International Instrumentation and Measurement Technology Conference (I2MTC).

[41] McGill W.J. (1954). Multivariate information transmission. Psychometrika.

[42] Jakulin A., Bratko I. Quantifying and Visualizing Attribute Interactions: An Approach Based on Entropy. http://arxiv.org/abs/cs.AI/0308002.

[43] Bell A. The co-information lattice. Proceedings of the 4th international symposium on Independent Component Analysis and Blind Source Separation (ICA 2003).

[44] Naghibi T., Hoffmann S., Pfister B. (2015). A Semidefinite Programming Based Search Strategy for Feature Selection with Mutual Information Measure. IEEE Trans. Pattern Anal. Mach. Intell..

[45] Goldfarb D., Liu S. (1990). An *O*(*n*^3^*L*) primal interior point algorithm for convex quadratic programming. Math. Programm..

[46] Lofberg J. YALMIP: A toolbox for modeling and optimization in MATLAB. Proceedings of the 2004 IEEE International Symposium on Computer Aided Control Systems Design.

[47] MOSEK-ApS (2017). The MOSEK Optimization Toolbox for MATLAB Manual. http://docs.mosek.com/8.0/toolbox/.

[48] Shen K.Q., Ong C.J., Li X.P., Hui Z., Wilder-Sniith E.P.V. (2007). A feature selection method for multilevel mental fatigue EEG classification. IEEE Trans. Biomed. Eng..

[49] Vedaldi A., Gulshan V., Varma M., Zisserman A. Multiple Kernels for Object Detection. Proceedings of the 2009 IEEE 12th International Conference on Computer Vision.

[50] Ma A.J., Yuen P.C., Lai J.H. (2013). Linear Dependency Modeling for Classifier Fusion and Feature Combination. IEEE Trans. Pattern Anal. Mach. Intell..

[51] Gehler P., Nowozin S. On Feature Combination for Multiclass Object Classification. Proceedings of the 2009 IEEE 12th International Conference on Computer Vision.

[52] Zarjam P., Epps J., Lovell N.H. (2015). Beyond Subjective Self-Rating: EEG Signal Classification of Cognitive Workload. IEEE Trans. Auton. Ment. Dev..

